# Nonsurgical Treatment Options for Basal Cell Carcinoma

**DOI:** 10.1155/2011/571734

**Published:** 2011-01-09

**Authors:** Mary H. Lien, Vernon K. Sondak

**Affiliations:** ^1^Department of Dermatology and Cutaneous Surgery, University of South Florida College of Medicine, 12901 Bruce B. Downs Boulevard, Tampa, FL 33626, USA; ^2^Cutaneous Oncology Program, H. Lee Moffitt Cancer Center and Research Institute, 12902 Magnolia Drive, Tampa, FL 33612, USA; ^3^Departments of Oncologic Sciences and Surgery, University of South Florida College of Medicine, 12901 Bruce B. Downs Boulevard, Tampa, FL 33626, USA

## Abstract

Basal cell carcinoma (BCC) remains the most common form of nonmelanoma skin cancer (NMSC) in Caucasians, with perhaps as many as 2 million new cases expected to occur in the United States in 2010. Many treatment options, including surgical interventions and nonsurgical alternatives, have been utilized to treat BCC. In this paper, two non-surgical options, imiquimod therapy and photodynamic therapy (PDT), will be discussed. Both modalities have demonstrated acceptable disease control rates, cosmetically superior outcomes, and short-term cost-effectiveness. Further studies evaluating long-term cure rates and long-term cost effectiveness of imiquimod therapy and PDT are needed.

## 1. Introduction

Basal cell carcinoma (BCC) remains the most common cutaneous malignancy in Caucasians, with as many as 2 million new cases expected to occur in the United States in 2010 [1–6]. Although surgical excision remains the standard treatment for BCC, nonsurgical modalities also have achieved acceptable cure rates [2]. Several factors may influence the decision to pursue nonsurgical alternatives, including lower overall costs and more cosmetically acceptable outcomes. In this paper, we will discuss two nonsurgical modalities for BCC treatment, topical imiquimod therapy, and photodynamic therapy.

## 2. Topical Imiquimod Therapy

Imiquimod (1-(2 methylpropyl)-1 H-imidazo (4, 5c) quinolin-4 amine) is an immunomodulator which stimulates the innate immune response via the upregulation and release of cytokines such as IFN-alpha, IL-6, and TNF-alpha as well as upregulating natural killer cell activity and upregulating nitric oxide secretion from macrophages [7, 8]. Imiquimod also upregulates the cell-mediated immune response through indirect stimulation of the Th1 cytokine IFN-gamma and activation of Langerhans cells to promote antigen presentation [9]. In vitro, imiquimod induces cytokine production via activation of Toll-like receptor 7 (TLR7), which in turn activates nuclear factor kappa B to stimulate production of IFN-alpha and cytokines IL-12 and IL-18. These cytokines then induce IFN-gamma production by naïve T cells, resulting in a Th1-type immune response. Thus, both IFN-alpha and IFN-gamma impart antiviral and antitumor activity to imiquimod. In addition, by stimulating cytotoxic T lymphocytes, IFN-gamma provides long-term immune memory. In essence, imiquimod stimulates both the innate and the cell-mediated arms of the immune system [10]. 

Imiquimod has been approved in the United States by the FDA for the treatment of superficial BCC (sBCC) in immunocompetent adults with tumors >0.5 cm^2^ in area and <2 cm in diameter located on the trunk and extremities [11]. Previous studies have utilized imiquimod 5% cream with application schedules ranging from 5 days to 7 days per week for 6–12 weeks. Studies also have reported the use of topical imiquimod to treat other BCC subtypes such as nodular BCC (nBCC) and sclerodermiform BCC [12–18]. One study demonstrated that, in a 2-year followup of 55 patients treated with imiquimod, 41 (74%) had complete remission (4/4 sBCCs (100%); 7/8 nBCCs (88%) and 30/43 infiltrative BCCs (70%)) [18]. In addition, imiquimod has also been explored as adjuvant therapy for patients after undergoing surgical treatment for nodular and superficial BCC [19–21]. 

Karve et al. recently compiled a summary of clinical trials evaluating the efficacy of imiquimod 5% cream in the treatment of sBCC, with the primary goal being the clearance of the tumor as detected by clinical and/or histological examinations [22]. Data from large randomized clinical trials demonstrated imiquimod 5% cream to be statistically superior to placebo in treating sBCC [22]. The clearance rates ranged from 75%–80.8% for patients treated 5 days/week and 73%–87.1% for patients treated 7 days/week. However, these clearance rates are significantly lower than those rates achieved by surgical methods [10]. 

No placebo-controlled, double-blind trials have evaluated the long-term sustained clearance of BCC after imiquimod therapy by utilizing an excisional biopsy of the treated site for histologic examination to document tumor eradication [22]. However, two phase 3 clinical trials, conducted in Europe and Australia/New Zealand, were designed to evaluate long-term clearance and both demonstrated similar initial clearance rates [23, 24]. For both of these studies, the estimated 2-year clearance was calculated by multiplying the initial 12-week clearance rate with the estimated nonrecurrence rates obtained from the life-table method [22]. Thus, 79.4% and 88.7% of the imiquimod recipients, respectively, were reported to be tumor-free two years posttreatment [23, 24].

In all the studies evaluating the efficacy of imiquimod 5% cream in the treatment of sBCC, over 50% of patients reported at least 1 adverse event during the study period [22]. The most frequently reported adverse event was a local application site reaction, such as itching, irritation, burning, tenderness, and hypopigmentation [22]. Patient compliance was defined either as situations in which patients applied at least 80% of the required dose or in which patients completed the prescribed dosing schedule [22]. The compliance of patients to the imiquimod therapy ranged from 60% to 98% [13, 18, 25]. 

Although surgical procedures such as surgical excisions, Mohs micrographic surgery, and curettage and electrodessication (C&E) comprise the current mainstay of surgical treatments of BCC, these procedures do not ensure 100% eradication of the tumor [6, 26]. Nodular BCC and sBCC are typically treated with surgical excision and C&E procedures. Furthermore, while C&E is frequently utilized to treat small tumors <1.5 cm in diameter, Mohs micrographic surgery addresses large tumor sizes >2 cm [22]. A range of 5-year recurrence rates after surgical excision have been reported to be from 0.7% to 10.1%, while the recurrence rates after C&E range from 3.3 to 7.7% [22]. In fact, the incidence of incomplete excision varies from 4.7% to 10.8% [27–29]. The purpose of employing imiquimod as adjunctive therapy for surgical excisions is twofold: to remove any residual tumor and to improve the cosmesis of the wound [21, 26, 30–34]. The inflammation induced by imiquimod may aid in the clearance of BCC [30]. Furthermore, imiquimod has been demonstrated to improve photoaging skin by increasing fibroplasia, decreasing elastosis, and restoring pigment [35]. 

In one study, topical imiquimod 5% cream after C&E of nodular BCC (*n* = 10) daily for 1 month resulted in fewer patients with residual tumor present at 8 weeks (10%) compared to control patients (40%). Furthermore, another larger study (*n* = 34) evaluated the efficacy of imiquimod 5% cream following curettage only of nodular BCC on the trunk and extremities [32]. 94% of the treated lesions showed histologic eradication of BCC. An earlier analysis revealed eradication rates to be as high as 96% after 3 years [21]. Therefore, the regimen of curettage with adjuvant imiquimod therapy may be considered to be an alternative treatment option of nodular BCC that additionally provides cosmetic advantages. 

There are very few studies comparing the cost-effectiveness of surgical treatment versus topical imiquimod therapy for sBCC. One 2007 study estimated the mean cost per patient treated with imiquimod 5% therapy (5 times/week for 6 weeks) to be lower than that of a patient treated with surgical excision for a single sBCC lesion less than 2 cm in diameter (€621 versus €676, resp.) [36]. However, one limitation of the study was that the costs associated with treating initial treatment failures or recurrences during long-term followup were not addressed. Another study compared the cost-effectiveness of imiqumod therapy versus surgical treatment of sBCC and reported that although imiquimod was more cost-effective in the short term (total costs: imiquimod €585 versus surgery €663), it was more expensive in the long term (total costs: imiquimod €1471 versus surgery €1322) [37]. However, the study limitation is that there were no calculated estimated efficacies of imiquimod therapy provided. Sverre et al. contrasted the cost-efficacy of imiquimod to the broad category of standard-of-care treatments (surgical excision, cryosurgery, or photodynamic therapy (PDT)) for sBCC [38]. The cost of imiquimod therapy was higher (by €16) than that of the latter category. Imiquimod however was found to be more cost-effective than PDT treatment, and was demonstrated to have a better outcome, albeit higher cost, than cryosurgery. To date, the long-term cost-effectiveness of imiquimod and its impact on the quality of life of patients with sBCC has not been comprehensively studied.

## 3. Photodynamic Therapy

BCC remains the most frequent oncologic application of PDT [39–41]. Although first described roughly a century ago, PDT did not spawn careful clinical investigation until the 1960s. Subsequently, PDT has been utilized in a wide variety of conditions, ranging from oncologic entities such as BCC and mycoses fungoides, to acne and psoriasis [42]. 

Photodynamic therapy utilizes oxygen radicals generated from a photoactive molecule to achieve a therapeutic tissue response. This photochemical reaction requires a photoactivating light, a photosensitizer, tissue oxygen, and a target cell. In this process, photon energy is converted to oxygen singlets, which then mediate target cell injury and death. Photosensitizers may be administered systemically or topically (as a prophotosensitizer). The mode of delivery determines tissue localization. 

Systemic photosensitizers, whether delivered via oral or intravenous routes, are comprised of large intact and lipophilic macrocytes which avidly bind to serum lipoproteins, chiefly the low-density lipoproteins (LDLs). These photosensitizers include porfimer, benzoporphyrin derivative monoacid ring A (BPD-MA), metatetrahydroxyphenylchlorin (mTHPC, temoporfin), and tin ethyl etiopurpurin (SnET2) [42]. In addition, tissue macrophages and tumor cells retain these photosensitizers, which contribute to the long-lasting photosensitivity that persists after their administration. In contrast, the prophotosensitizers are low-molecular weight, hydrophilic and are topically administered onto the affected skin surface. These include 5-delta-aminolevulinic acid HCL (ALA) and methyl-esterified ALA (mALA). In the latter case, the stratum corneum of the skin is typically removed via superficial curettage prior to mALA topical application. After absorption, mALA is then metabolized to ALA, upon which it is easily absorbed through the cell membrane. 

The gold standard of a photosensitizer is one which maintains reduced tissue retention so that there is a decreased risk of prolonged systemic photosensitivity. Protoporphyrin IX (PpIX) maintains a high tissue retention due to its hydrophobic qualities. However, the administration of a prophotosensitizer, such as ALA, enables cells to produce PpIX themselves for photoactivation. Since 1987, BCC, actinic keratoses (AKs), and squamous cell carcinoma (SCC) have been treated with topical ALA and red light [43, 44]. PDT received FDA approval in 1999 for the treatment of AKs utilizing topical ALA plus blue light. 

The effectiveness of PDT responses depends upon the tissue localization of photosensitizers. Following drug administration, adequate time must be allowed for proper distribution of the photosensitizers to the target tissues. Ideally, photoactivation should occur when the photosensitizer concentration in the target tissue exceeds that in the normal tissue in order to achieve the maximum efficacy of PDT. 

In order to minimize tissue penetration of ultraviolet (UV) light and the subsequent risk of incurring skin cancers, the PDT wavelength utilized for treatment must be longer than those in the UV spectrum. Thus, the PDT wavelength range is between 400 and 800 nm. Increased tissue penetration correlates with increased wavelength; therefore, blue light provides adequate penetration for treating epidermal lesions. In contrast, dermal lesions necessitate deeper penetrating red light that must correspond with one of the photosensitizer's absorption peaks. Visible light from noncoherent or coherent (laser) sources is used. The two types of light include low-power, continuous wave, and nonthermal versus high-power, pulsed and photothermal. The latter light type utilizes biologic chromophores (i.e., melanosomes, blood vessels, etc.) to amplify tissue injury. 

In order to successfully treat cutaneous malignancies, and premalignancies with PDT, the minimum “photodynamic dose,” or threshold of injury, must be achieved in order to induce a degree of cell destruction which cannot be reversed through inherent cell repair processes. Consequently, PDT induces intense inflammation through the release of cytokines, chemokines and other immunologic proteins by the injured and apoptotic cells. PDT has also been demonstrated to act as a biologic response modifier [45]. In addition to damaging target cells directly, PDT, through upregulated cytokine production, enhances the innate and adaptive immune responses in immunocompetent individuals [45]. 

Although PDT is commonly utilized to treat BCC, there exist few studies which directly compare the efficacy of PDT against the standard treatment modalities such as surgical excision, Mohs surgery, or cryosurgery. There is evidence that systemic porphyrin-based PDT induces a complete response in 80% to 100% of BCC cases, but no long-term followup was performed [46–48]. Selected studies utilizing systemic photosensitizers (porfimer sodium, BPD-MA and meta-tetrahydroxyphenylchlorin [mTHPC]), and red light were analyzed [49–52]. Although good response rates were achieved (78% to 88% range), significant adverse effects included generalized photosensitivity, facial edema, and severe pain during treatment and posttreatment [49–52]. Furthermore, higher doses of photosensitizer and higher light fluences were associated with better response rates, and mTHPC usage necessitated much shorter illumination periods [42]. 

There is a desire to minimize the use of systemic photosensitizers due to the aforementioned risks associated with prolonged generalized photosensitivity. Consequently, the topical photosensitizers (ALA, mALA) have gained favor due to their decreased adverse effects and their convenient use. Early studies reported a 79% complete response rate at 3 months for 300 sBCC treated with ALA 20% (3–6 hours) using filtered red (600+ nm) light [43, 44]. Subsequent studies utilizing topical ALA or mALA and red light demonstrate superior response rates for sBCC than for nodular BCC [53–56]. One study compared the therapies of mALA-PDT with surgical excision (>5 mm margins) of nodular BCC and demonstrated complete response rates at 1 year of 83% and 96%, respectively [55]. 

Topical photodynamic therapy is generally well tolerated [42]. Immediate effects after treatment include erythema and slight edema. After a few days, crusts and superficial erosions may be noted, and ulcerations are uncommon [57]. The most common acute side effect experienced by patients is pain, which has been described as a burning, stinging, or prickly sensation in the ALA-treated sites during light exposure [58]. The mechanism is unclear, but hyperthermia related to the presence of a reactive singlet oxygen has been implicated. 

The pain associated with ALA-PDT is also related to the site and size of the treated area [57]. The treatment of facial and scalp lesions have been noted to be associated with greater incidences of pain [57]. In instances involving the treatment of large surface areas, local anesthesia is impractical for PDT treatment of extensive regions. Case reports have documented the treatment of large surface areas (up to 22% BSA) over 3–6 hours under general anesthesia in these patients [57, 58]. In children, general anesthesia has been demonstrated to be safer than conscious sedation [57]. Pain management options incude local anesthesia, premedication with opiod analgesics, cooling fans or spraying the treated areas with water [57]. Cold air analgesia has been found to be effective in some cases; however, tetracaine gel, capsaicin cream, and morphine gel have been found to be ineffective [58]. 

Potential disadvantages of topical PDT in the treatment of BCC include insufficient drug delivery at depth or insufficient light delivery at depth [38]. One study demonstrated that although 69% of the topical ALA-treated tumors (BCC, SCC in situ, invasive SCC) were clinically clear, only 46% of the treated tumors were histologically clear due to subclinical extension of the tumor [59]. This finding suggests that cancer recurrences following PDT may result from inadequate penetration of topical ALA into the deep dermis. The aggressive subtypes of BCC, including morpheaform BCC, would demonstrate the least drug penetration due to their intact stratum corneum. 

Attempts to enhance tissue penetration of ALA and to improve the cure rates of BCC treated with topical ALA PDT include curettage of the tumor site immediately prior to ALA application; pretreatment with dimethylsulfoxide (DMSO); prolonged ALA application (up to 48 hours); intralesional ALA injection [42]. In addition, ALA has been compounded with ethylenediaminetetraacetic acid (EDTA) or desferrioxamine to enhance PpIX formation. These protocols have demonstrated improvement in long-term cure rates [42]. 

To address the drug penetration issue, modifications in the ALA administration protocols have been utilized. For example, studies using abbreviated systemic ALA administration have used a single ALA dosage, or fractionated oral ALA dosage (60 mg/kg delivered in 20 mg/kg increments) and fractionated red light (600–650 nm; 50 or 100 J/cm^2^) to reduce the duration of generalized photosensitivity and to circumvent transient liver enzyme elevation [42]. 

Topical mALA is more lipophilic than ALA and demonstrates increased tissue penetration. In a retrospective review (*n* = 350) of the treatment of BCC with mALA-PDT, the initial complete response was confirmed years later (mean 35 months) in 89% of the treated sites, with 11% recurrences (both morpheaform BCCs recurred after treatment) [56]. Overall, the existing data suggest that mALA is an effective agent with PDT therapy and that nodular BCC may be more responsive to mALA than to ALA [42]. 

In a study by Caekelbergh et al., cosmetic outcome was defined as excellent (no scarring, atrophy or induration and no or slight occurrence of redness or change in pigmentation compared to surrounding skin); good (no scarring, atrophy or induration, but moderate redness or pigmentary change compared to surrounding skin) fair (slight to moderate occurrence of scarring, atrophy or induration), or poor (marked occurrence of scarring, atrophy or induration) [60]. The cosmetic outcomes from PDT were deemed to be more favorable than those from standard surgical therapy (margins >5 mm) or cryotherapy [42]. Due to its ability to treat large body surface areas with a single administration and with minimal scarring, topical PDT is ideal to treat patients with familial cancer syndromes including Gorlin syndrome (nevoid basal cell carcinoma syndrome). 

The cost-effectiveness of PDT using mALA for sBCC was addressed in a prospective, observational study (*n* = 90 patients) by Caekelbergh et al. [60]. The analysis revealed a complete clinical response rate of 89% for sBCC 6 months after the first mALA-PDT treatment. The study also found that mALA-PDT was more cost-effective than surgical excision in the treatment of sBCC [60]. 

## 4. Conclusion

In summary, imiquimod therapy and PDT have been demonstrated to achieve acceptable short-term cure rates of BCC. Clearance rates of 73% to 87.1% have been reported in patients with sBCC treated with imiquimod 5% cream. Imiquimod is also an effective adjuvant therapy in patients with BCC treated with surgical treatments. Topical photosensitizers are favored for use in PDT due to the lower risk of adverse effects, convenient use, and the ability to treat large surface areas. Topical ALA-PDT has been demonstrated to achieve a clearance rate of 79% for sBCC. In addition to treating sBCC, topical mALA-PDT also has been shown to be effective in treating nodular BCC due to its deeper tissue penetration. 

Due to their improved cosmesis, cost-effectiveness, and minimally invasive nature, these nonsurgical alternatives may be considered for treatment of appropriate tumors, especially sBCC. Long-term recurrence rates and safety of imiquimod use and PDT use have yet to be determined. Further studies evaluating the long-term parameters of efficacy, safety, and cost-effectiveness of imiquimod therapy and PDT are warranted. 
